# Diagnosis of Indian Visceral Leishmaniasis by Nucleic Acid Detection Using PCR

**DOI:** 10.1371/journal.pone.0019304

**Published:** 2011-04-29

**Authors:** Pankaj Srivastava, Sanjana Mehrotra, Puja Tiwary, Jaya Chakravarty, Shyam Sundar

**Affiliations:** Infectious Disease Research Laboratory, Department of Medicine, Institute of Medical Sciences, Banaras Hindu University, Varanasi, India; Université Pierre et Marie Curie, France

## Abstract

**Background:**

PCR based diagnosis for Visceral Leishmaniasis (VL), despite numerous published primers, remains far from being applied in the field. The present study was planned to design a *Leishmania* specific diagnostic assay and to evaluate its sensitivity and specificity on a sample size, which to the best of our knowledge is the largest ever screened in one study.

**Methods:**

*Leishmania* specific primers were developed using 18S rRNA gene and their sensitivity was evaluated on 500 parasitologically confirmed patients with VL and 25 Post Kala-azar Dermal Leishmaniasis (PKDL) patients. Specificity was calculated on 250 healthy endemic controls, 250 healthy non endemic controls and 250 non leishmanial diseases like malaria.

**Results:**

Our PCR assay had a sensitivity of 87.8% (95%CI: 84.1–89.8) using 200 µL of patient's peripheral-blood. Specificity was absolute in non-endemic healthy controls and in subjects with different diseases while in endemic controls it was 84% (95%CI: 78.9–88.0). Its overall specificity was 94.6% (95%CI-92.8–96.1).

**Conclusions:**

The PCR assay developed is sensitive enough to detect the 18S rRNA gene in an amount equivalent to a single parasite or less in a one million human cell environment. The high sensitivity of this PCR diagnostic test with relatively non-invasive peripheral blood sampling method opens up the possibility of its deployment in field for the routine diagnosis of VL.

## Introduction

The gold standard for the diagnosis of Visceral Leishmaniasis (VL) is demonstration of parasites (amastigotes) in giemsa-stained smears prepared from splenic or bone marrow aspirates [1]. Splenic smears have high sensitivity, but are associated with a risk of serious/fatal haemorrhage, while bone marrow smears have low sensitivity and the procedure is painful [2]. Serological tests like the direct agglutination test (DAT), rK39 immunochromatographic tests and the Indirect Immunofluorescent Antibody Test all have high sensitivity but cannot discriminate between past and current infections [2], [3], [4]. Molecular diagnosis exploiting PCR combines several advantages; it is minimally invasive, has a high sensitivity and specificity, is capable of identifying relapses and reinfections in treated VL patients, and can provide species identification [5], [6]. Many different PCR assays have targeted conserved and variable regions of kDNA minicircles [7], [8], [9], [10], [11], genomic DNA, splice leader mini-exon (SLME) [12], telomeric repeats [13], rRNA gene [14] and gp63 [15] for the detection of parasites directly from human tissues. However, despite countless reports describing PCR in VL diagnosis, none of these assays are used as a diagnostic tool in clinical setting. The aim of this study was to develop a PCR assay, with a view to assess its robustness in large sample size, and develop a suitable field version for studies performed in endemic regions.

## Materials and Methods

### Study Site, Patients and Controls

The study was approved by the Institutional Ethical Committee of Institute of Medical Sciences (IMS), Banaras Hindu University (BHU), Varanasi and a written informed consent was obtained from all subjects. The study was conducted between January 2007 and December 2009. Parasitologically confirmed VL patients (n = 500) were recruited from the field site Kala Azar Medical Research Centre (KAMRC) at Muzaffarpur, Bihar and Sir Sundarlal Hospital, Banaras Hindu University, Varanasi. Twenty five Post Kala-azar Dermal Leishmaniasis (PKDL) patients were also included in the study. Controls included 250 healthy controls belonging to endemic areas with active transmission of VL (EHC), 250 healthy controls coming from regions not endemic for VL (NEHC), and 250 patients with common infectious diseases like Malaria, TB (Different Diseases) were also included in the study to evaluate the specificity of the assay.

### Sample collection, storage, and DNA preparation

Peripheral blood samples (1–2 ml) were collected in heparin vacutainers and stored at 4°C. The samples were transported at 4°C from KAMRC, Muzaffarpur to BHU where they were processed within a week. DNA was isolated from 200 µl peripheral blood using QIAamp Blood DNA mini kit (Qiagen) according to the manufacturer's instructions. All the samples were quantified using picodrop spectrophotometer (Picodrop Limited, UK), aliquoted in triplicates to avoid DNA damage during repeated freeze thawing and stored at −20°C. DNA preparations were also made from *Leishmania donovani* promastigotes using the QIAamp DNA Mini Kit (Qiagen). The parasitic DNA was diluted in 10 fold serial dilutions starting from 1000 ng upto .001 pg.

### Preparation of spiked samples

Freshly drawn peripheral blood from a healthy volunteer was used to prepare spiked samples in triplicate for each dilution. *L. donovani* promastigotes ranging from 10,000 to 100 parasites were spiked in 200 µl of blood in a 10 fold dilution. Counting of parasites was performed using a haemocytometer. For spiking 10 and 1 parasite, the hanging drop method was used. Pre- dilution of the parasites in 2 ml cRPMI media was made and a microdrop of it was put on the cavity slide using sterile glass micropipette. The slide was sealed with the coverslip and sealing media to prevent drying of the drop. Several dilutions were tested till we could see a single parasite in the drop under an inverted microscope which was then spiked into 200 µl of blood. DNA from these spiked samples was isolated using QIAamp DNA Blood Mini Kit (Qiagen) and quantified in a similar way as described above.

### Primer designing

**Figure 1:**
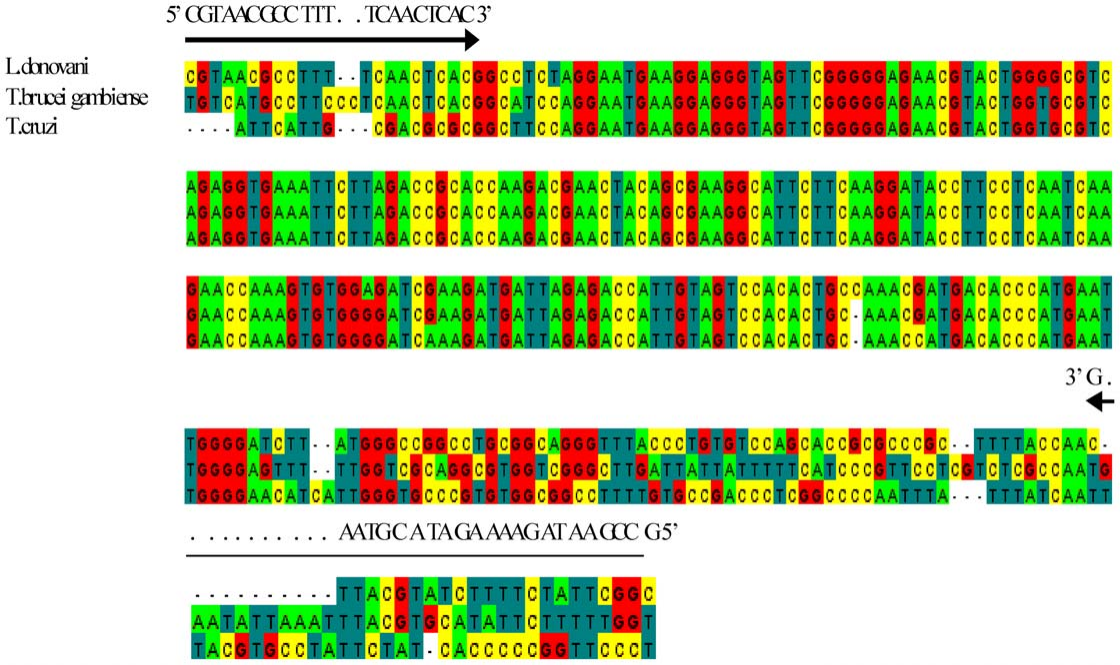
Sequence alignment and primer designing. Alignment of the DNA sequence within the 18S rRNA gene of *Leishmania donovani* (*L.d.*; GenBank accession no. X07773), *Trypanosoma brucei gambiense* (*T.b.*; GenBank accession no. AJ009141), and *Trypanosoma cruzi* (*T.c.*; GenBank accession no. AF303660). The forward (BHUL18SF) and reverse (BHUL18SR) primers are indicated by arrows.

Since primers are of crucial importance to the success of any diagnostic PCR assay, a rigorous bioinformatics analysis was done. The 18S rRNA gene was chosen as the target region because it is a multicopy gene and is present in 50–200 copies per *Leishmania* genome. 18S rRNA gene sequences from *L. donovani* (GenBank Accession ID X0773), *T b. gambiense* (AJ009141) and *T. cruzi* (AF303660) were aligned using ClustalW software version 2 (http://www.ebi.ac.uk/Tools/clustalw2) (Figure 1) and primers were designed using the most stringent conditions allowed in the software package against the conserved regions by using OLIGO4 primer analysis software. At least 3–4 mismatches at 3′ end of both forward and reverse primers were ensured within the aligned sequences to ascertain that the primers do not show any cross reactivity with closely related trypanosomatids. The forward primer, designated as BHUL18SF (5′ CGTAACGCCTTTTCAACTCAC 3′) and reverse primer as BHUL18SR (5′ GCCGAATAGAAAAGATACGTAAG 3′), amplify a region of 311 bp. The specificity of the primers was examined using the BLAST programme at NCBI site. In silico PCR was performed using UCSC genome browser (http://genome.ucsc.edu/) to be confident that the primers do not show any cross reactivity with human sequences. The primers were synthesized by Metabion, Germany.

### PCR optimization

PCR was first optimised using parasitic DNA and then on clinical samples. The reaction mixture (25 µL) contained 1× Taq Polymerase Buffer (Promega), 1.5 mM MgCl_2_, 200 µM concentration of each deoxynucleotide triphosphate (dNTP), 5 pmole of each primer, 1.5 U of Taq DNA Polymerase (Promega) and 150 ng of template. The annealing temperature of the primers was determined by setting up a temperature gradient PCR (Veriti, ABI, Foster City, USA) in increments of 2°C starting from 52°C. The reaction mixture using primer BHUL18SF/R was amplified in ABI Gene Amp PCR System 9700 (ABI, Foster City, USA) with heated lid option at 95°C for 4 min followed by 40 cycles, each consisting of 45 secs at 94°C, 45 secs at 62°C, and 1 min at 72°C, and a final extension step of 5 min at 72°C. Products were analysed by electrophoresis on 1.5% agarose (Thermo Scientific, Mol Bio Grade) gel, containing ethidium bromide (0.5 µg/ml in TAE buffer), 0.04 M Tris acetate, 0.001 M EDTA and photographed under UV illumination and bands quantified by using Gel documentation software (Alfa Innotech Corporation, San Leonardo, CA, USA). For each experiment, multiple numbers of negative controls were included to check for possible contamination.

### Sequencing reaction

The PCR amplification products from culture isolates and clinical samples from patients with VL and PKDL were cloned into the pGEMT-Easy vector system (Promega). The amplified PCR product was purified from gel using Sigma Gel elute columns. Sequencing PCR was conducted using a Thermo Sequenase premixed ABI BigDye Terminator 3.1 cycle sequencing kit (Applied Biosystems, Inc., Foster City, CA) according to the manufacturer's instructions under following conditions: first heating at 96°C for 1 min and then 30 cycles consisting of 96°C for 10 sec and 50°C for 10 sec, 60°C for 30 sec and 60°C for 4 min. After ethanol precipitation and purification, the sequencing samples were loaded to ABI3130 genetic analyser. Sequence analysis was done by Chromas software.

## Results

### Detection limit of optimised PCR assay with BHUL18S primer

**Figure 2:**
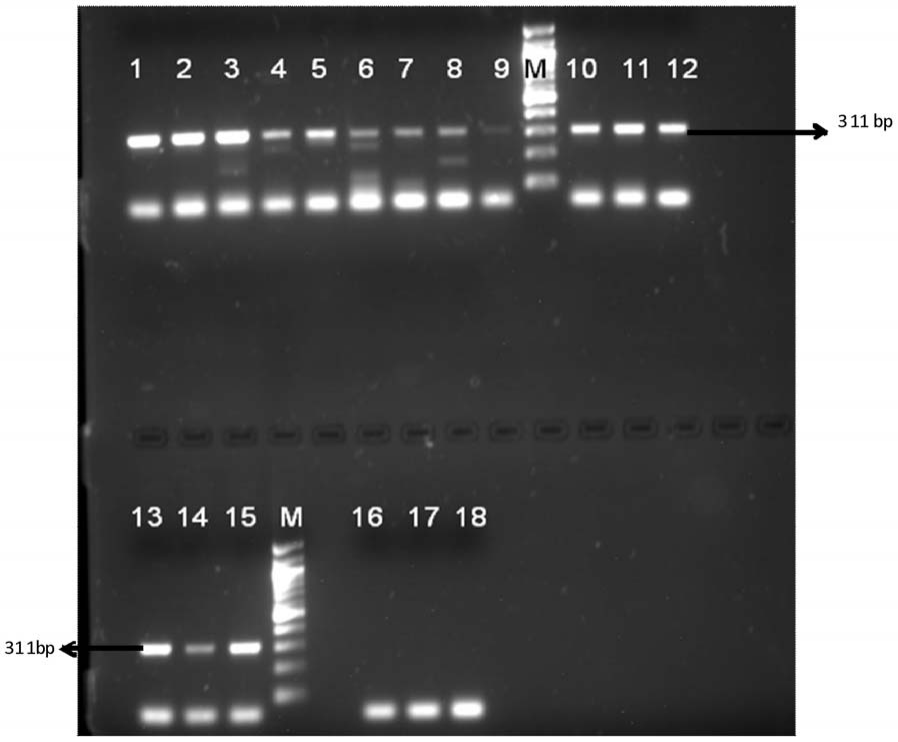
PCR result for spiked samples. Lane 1–3: 10,000 parasites, Lane 4–6: 1000 parasites, Lane 7–9: 100 parasites, Lane 10–12: 10 parasites, Lane 13–15: 1 parasite, Lane 16–18: No Template controls (NTC), M = 100 bp marker.

Sensitivity analysis of primer BHUL18S with serially diluted parasitic DNA showed that the lower limits of detection of developed PCR assay was 100 fg. The results with spiked blood samples showed that the assay was sensitive enough to detect down to 1 parasite spiked per 200 µl of blood (Figure 2).

### Evaluation in Clinical Samples

**Figure 3:**
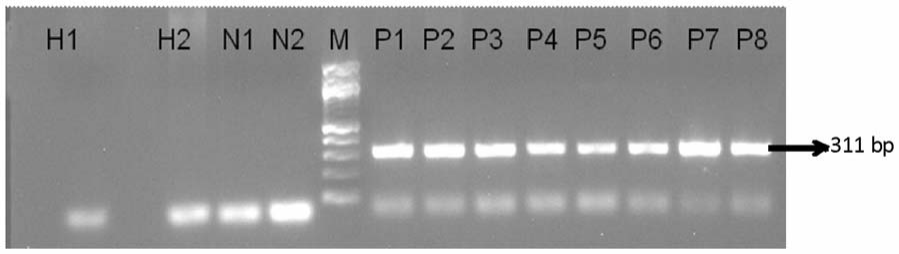
PCR results in clinical samples. Lanes H1and H2: Non-endemic healthy controls, Lane N1 and N2: negative controls, Lanes P1 to P8: VL patients, M = 100 bp marker.

Of the 500 parasitologically confirmed clinical samples, 439 were PCR positive with BHUL18S primer (Figure 3). The test provided successful diagnosis of VL with 87.8% (95% CI: 84.1–89.8) sensitivity using patient's whole peripheral blood (Table 1). In endemic controls, the specificity was 84% (95% CI: 78.9–88%) (Table 2). None of the 250 nonendemic controls were positive and thus, the specificity in this group was 100% (95% CI: 98.5–100). The data from the different disease group also showed 100% (95% CI: 98.5–100) specificity as none of the 250 non-leishmanial disease samples were PCR positive. The overall specificity was 94.6% (95% CI: 92.8–96.1).

**Table 1:** Sensitivity of the PCR assay in confirmed VL cases.

Subjects	N	Positive N (sensitivity in %)	Negative	95% CI
VL Patients	500	439 (87.8)	61	84.1–89.8%
PKDL Patients	25	19 (76.0%)	6	

**Table 2:** Comparative specificity in different control groups.

Subjects	N	Positive	Negative	% Specificity (95% CI)
Endemic Controls	250	40	210	84(78.9–88%)
Healthy Controls	250	0	250	100 (98.5–100%)
Different Disease (Non VL)	250	0	250	100(98.5–100%)
Total	750	40	710	94.6 (92.8–96.1%)

Nucleotide BLAST analysis after sequencing of PCR products obtained from clinical samples, as well as from parasite isolates, revealed that there was 100% identity with the 18S rRNA gene of *L. donovani* and no identity to human rRNA or *Plasmodium sp*, *Mycobacterium* etc.

## Discussion

The study was initiated to develop, as well as evaluate the sensitivity and specificity of the designed primers in a PCR assay in peripheral blood samples from VL patients. To our knowledge, this study has validated the largest number of patients and controls for diagnosis of VL through PCR. We looked at the critical points affecting the success of a diagnostic PCR and standardized and optimized the protocols leading to development of a PCR assay which may be used for routine diagnosis in endemic regions of Bihar. Currently the molecular diagnosis of VL solely relies on PCR based assays of various throughputs, extensively reviewed by Reithinger et al [16]. Recently chromatography based Oligo-C test and NASBA have been introduced [17] and their sensitivity and specificity was determined in Sudan [18], East Africa [19] and Kenya [20] in clinical samples from VL patients. However, none of them are yet an option for diagnosis in Indian clinical settings because of the lack of standardisation and the insufficient sample size included in the experiments. Our study, which involves normal qualitative PCR in diagnosis of VL, has been validated on a large sample size wherein lies its significance. The PCR reaction conditions were thoroughly optimized and consistent results were obtained. The rRNA gene which has a copy number of 50–200 was chosen and we were able to reach high sensitivities of 87.8%. The 12.8% negative patients may have had very low circulating parasites in blood leading to negative PCR. In the clinical setting, if there is a negative PCR result and a high level of clinical suspicion, then other methods of detection such as splenic aspiration or serology could be used [21]. Since the sensitivity and specificity of any PCR assay depends on many factors, precautions were taken at each and every step starting from sample collection till DNA isolation. Strict measures like complete separation of pre PCR and post PCR regions, 3–4 negative controls with each set of experiment helped in achieving the high specifity level. Until now, apart from in research settings, in most endemic regions in Indian subcontinent, surrogate markers involving anti- rK39 rapid tests is being used for diagnosis, where up to 30% of endemic controls, and 2–3% of subjects with difference diseases like malaria or tuberculosis may test positive. Molecular based tests are more specific as in our study none of patients with other diseases tested positive. However, a 16% PCR positivity in healthy endemic controls was obtained. This reinvigorates the prevalence of asymptomatic individuals who harbor *Leishmania* parasites without developing clinical symptoms. This is a consistent finding with many different types of diagnostic test in the context of the endemic healthy population [22], [23]. Since undetected and untreated infections can continue the chain of transmission and can lead to serious long-term complications, tests to screen individuals for such asymptomatic infections might reduce disease transmission in the community and hence can be an added advantage of the developed assay. The developed assay is not species specific since SSU rRNA gene is highly conserved within *Leishmania* species. However, this is not a drawback since it is well established that *L. donovani* is the only causal agent of VL in Indian subcontinent.

Whether PCR will eventually replace the gold standard of parasite demonstration and serology based detection is a controversial question. The overall cost calculated for one PCR assay starting from DNA isolation in this study was minimal, only Rs 230 (less than $5) which is quite similar to cost of one rk39 strip. A basic molecular biology lab with minimum infrastructure; a thermocycler, a UV transilluminator and a gel electrophoresis unit are a onetime investment and if they can be provided, PCR could be performed in an endemic setting for routine diagnosis.

In conclusion, we report here the results of a PCR study with high sensitivity and specificity. The present study is robust as it was performed on a large sample size over two years with remarkable consistency. The next step would be to decentralize this technique to at least district level especially in the endemic areas where one PCR laboratory with capability to perform PCR could obviate the need of risky procedures like splenic aspirate and provide a more specific diagnosis than the currently used surrogate markers.
